# Peripheral blood immune landscape and NXPE3 as a novel biomarker for hypertensive intracerebral hemorrhage risk prediction and targeted therapy

**DOI:** 10.1002/imt2.70030

**Published:** 2025-04-11

**Authors:** Meng Zhang, Jingyuan Ning, Jing Liu, Yingying Sun, Ning Xiao, Haochen Xu, Jingzhou Chen

**Affiliations:** ^1^ State Key Laboratory of Cardiovascular Disease, Fuwai Hospital, National Center for Cardiovascular Diseases, Chinese Academy of Medical Sciences and Peking Union Medical College Beijing China; ^2^ State Key Laboratory of Common Mechanism Research for Major Diseases & Department of Medical Genetics, Institute of Basic Medical Sciences & School of Basic Medicine Chinese Academy of Medical Sciences & Peking Union Medical College Beijing China; ^3^ National Health Commission Key Laboratory of Cardiovascular Regenerative Medicine, Fuwai Central‐China Hospital, Central‐China Branch of National Center for Cardiovascular Diseases Zhengzhou China

## Abstract

We employed bulk RNA‐seq and scRNA‐seq techniques to analyze the immune dysregulation in patients with intracerebral hemorrhage (ICH). The study revealed that excessive inflammatory responses, neutrophil activation, and T‐cell dysfunction are the main characteristics of ICH. A multi‐machine learning framework was utilized to construct a predictive model for ICH risk in hypertensive patients. Molecular docking and simulation results demonstrated that NXPE3 is a potential therapeutic target, and dihydroergotamine (DHE) exhibits a strong binding affinity to NXPE3. In vivo experiments indicated that DHE can reduce the incidence of spontaneous ICH and modulate the immune‐inflammatory response. These results support DHE targeting NXPE3 as a potential therapeutic strategy for hypertension‐related ICH.

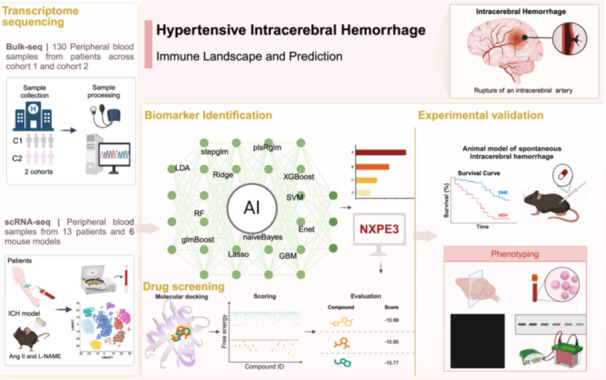

## ETHICS STATEMENT

All procedures were conducted in compliance with the relevant guidelines and regulations. This study received approval from the Human Ethics Committee of Fuwai Hospital (Approval No. 2016‐732, No. 2022‐1692) and adhered to the principles of Good Clinical Practice and the Declaration of Helsinki. Written informed consent was obtained from all participants or their legal representatives.

To the Editor,

Hypertensive intracerebral hemorrhage (ICH) represents a severe complication of high blood pressure (HBP), exhibiting high rates of morbidity and mortality and imposing significant societal and familial burdens [1, 2, 3]. Current diagnostic and therapeutic strategies are not sufficiently individualized and lack early risk prediction, limiting their effectiveness in identifying hypertensive individuals at risk of ICH [4]. Consequently, a more profound understanding of the underlying pathophysiological mechanisms, alongside the development of refined diagnostic tools and targeted intervention strategies, is critical for distinguishing hypertension from hypertensive ICH and formulating more effective preventive measures. Accumulating evidence underscores the pivotal role of immune and inflammatory responses in the progression of the disease [5, 6]. Immune modulation therapies, therefore, emerge as a promising strategy for both diagnosis and treatment.

Bulk transcriptome sequencing of peripheral blood from 130 hypertensive and hypertensive ICH individuals across two cohorts provided a comprehensive immunological profile. Additionally, single‐cell sequencing was performed on blood samples from hypertensive patients, individuals with cerebral microbleeds, and hypertensive ICH mouse models, with a particular focus on neutrophil transformation. A predictive model for ICH risk was developed based on neutrophil‐related genes and 134 machine‐learning frameworks. Through this approach, *NXPE3* was identified as a novel biomarker and therapeutic target, opening new avenues for clinical management. Targeting NXPE3 holds the potential to reduce ICH‐related mortality and enhance functional outcomes, thereby advancing the application of precision medicine in hypertensive ICH.

## RESULTS AND DISCUSSION

### Bulk and single‐cell transcriptomic sequencing reveal immune microenvironment dysregulation in the peripheral blood of patients with intracerebral hemorrhage

**Figure 1:**
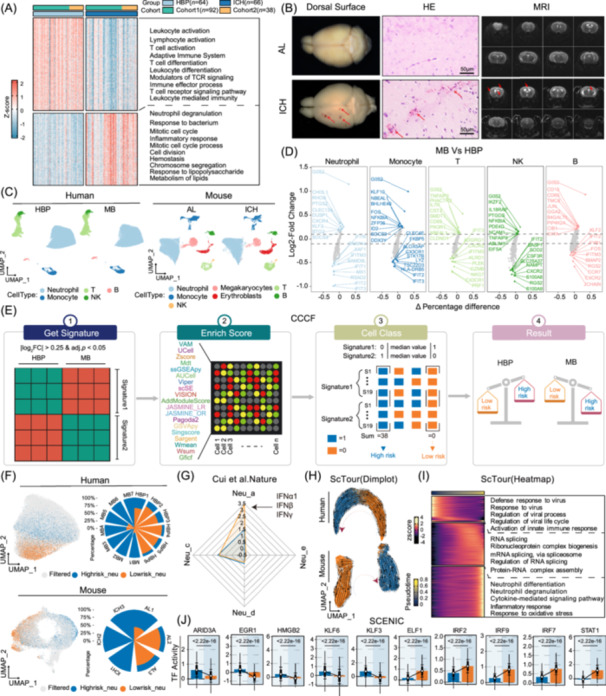
Transcriptomic sequencing reveals immunological features in the peripheral blood of patients with hypertensive intracerebral hemorrhage. (A) Heatmap and enrichment of differentially expressed genes (DEGs) in intracerebral hemorrhage (ICH) and high blood pressure (HBP) samples. (B) Representative images of the dorsal surface, hematoxylin and eosin (H&E) staining, and magnetic resonance imaging (MRI) in the mouse model. (C) The uniform manifold approximation and projection (UMAP) plot of single‐cell RNA sequencing (scRNA‐seq) annotation. (D) Differential analysis of cell types between microbleed (MB) and HBP in human data revealed transcriptional changes in each cell lineage in MB patients. The alterations in neutrophils and T cells were consistent with bulk transcriptome disturbances. (E) Cellular classification consensus framework (CCCF) workflow. Firstly, differential expression analysis of neutrophils in human single‐cell sequencing was performed using Seurat (v4.4.0) and the FindMarkers function. Genes with |log_2_ fold change| > 0.25 and adjusted *p* < 0.05 were classified as differentially expressed in the ICH group. Upregulated genes as signature1, and downregulated genes as signature2. Gene set scoring for each cell was done using the irGSEA package (v3.2.6), calculating scores for both signature1 and signature2. Cells with signature1 scores above the median were assigned 1, otherwise 0; for signature2, cells with scores above the median were assigned 0, otherwise 1. With 19 scoring algorithms, each cell received 38 scores—19 for signature1 and 19 for signature2. Cells were classified based on the sum of these scores: a total of 38 indicated high‐risk neutrophils, while 0 indicated low‐risk neutrophils. Cells with intermediate scores were filtered. (F) UMAP and proportion analysis of high‐risk and low‐risk neutrophils in human and mouse scRNA‐seq. Spatially, high‐risk and low‐risk cells exhibit distinct separation. (G) Low‐risk neutrophils exhibit elevated Neu_a characteristics compared to high‐risk neutrophils. (H) Pseudotime trajectory of human and mouse neutrophils. (I) Heatmap of pseudotime gene expression with stage‐specific enrichment. (J) Transcription factor activity between high‐risk and low‐risk cells.

Peripheral blood was collected from 130 hypertensive patients across two cohorts, followed by bulk transcriptome sequencing (Table S1). Post‐batch effect correction, the principal component analysis revealed a distinct separation between the ICH and HBP groups (Figure S1A). Differential expression analysis identified 1475 differentially expressed genes (DEGs) (log_2_FC| > 1, adj. *p* < 0.05) (Figure 1A). Upregulated DEGs were predominantly enriched in neutrophil degranulation and inflammatory response pathways, while downregulated genes were associated with leukocyte and T cell activation, suggesting neutrophil activation and T cell suppression. Additionally, scRNA‐seq was performed on peripheral blood from HBP and microbleed (MB) individuals, alongside AL and ICH mouse models (Figure 1B). Neutrophils were the most abundant cell type (Figures 1C and S1B), with only a slight increase in monocytes in the MB group (Figure S1C). These findings imply that alterations in cell composition are not a prominent characteristic of ICH. However, substantial transcriptional changes were observed in the MB group relative to HBP (|log_2_FC| > 0.25, adj. *p* < 0.05) (Figure 1D). Notably, shared transcriptional alterations across cell types included upregulation of *G0S2* and downregulation of *CXCR2* and *CX3CR1*. T cells exhibited downregulation of *NKG7*, *PRF1*, and *GZMB*, consistent with bulk transcriptomic findings. These alterations were similarly validated in mouse samples (Figure S1D).

Neutrophils associated with the high‐risk phenotype were analyzed using a novel cellular classification consensus framework (CCCF) (Figure 1E). Application of this framework to human scRNA‐seq data revealed a higher proportion of high‐risk neutrophils in patients with MB, contrasted with predominantly low‐risk neutrophils in patients with HBP, with uniform manifold approximation and projection demonstrating clear separation between these populations (Figure 1F). This finding was further corroborated by mouse scRNA‐seq and human bulk‐seq data (Figures 1F and S1E), confirming the CCCF's efficacy in accurately identifying high‐risk neutrophils associated with ICH. According to Cui et al. [7], neutrophils can differentiate into four distinct states under varying cytokine stimuli. Compared to low‐risk cells, high‐risk neutrophils exhibited diminished polarization toward the Neu_a state, suggesting a deficiency in IFNα1, IFNβ, and IFNγ stimulation within their immune microenvironment (Figure 1G). Further examination of Neu_a marker genes (*GBP2*, *HERC6*, *GBP5*, and *ISG15)* revealed significant upregulation in low‐risk neutrophils, with similar trends observed for *Gbp2*, *Herc6*, and *Isg15* in mouse samples (Figure S1F). Using the scTour method [8], the differentiation trajectory from low‐risk to high‐risk neutrophils was reconstructed and validated in mice (Figure 1H), demonstrating a stable process. Early differentiation stages were enriched in innate immune and viral defense pathways (Figure 1I). Mid‐differentiation stages showed enrichment in RNA splicing pathways. Terminal stages exhibited enrichment in neutrophil degranulation and inflammatory response. SCENIC [9] analysis revealed upregulated transcription factors (*ARID3A, EGR1, HMGB2, KLF6, KLF3*) and downregulated factors (*ELF1, IRF2, IRF7, IRF9, STAT1*) in high‐risk neutrophils (Figure 1J).

### Multi‐machine learning integration to build predictive models for ICH early warning

**Figure 2:**
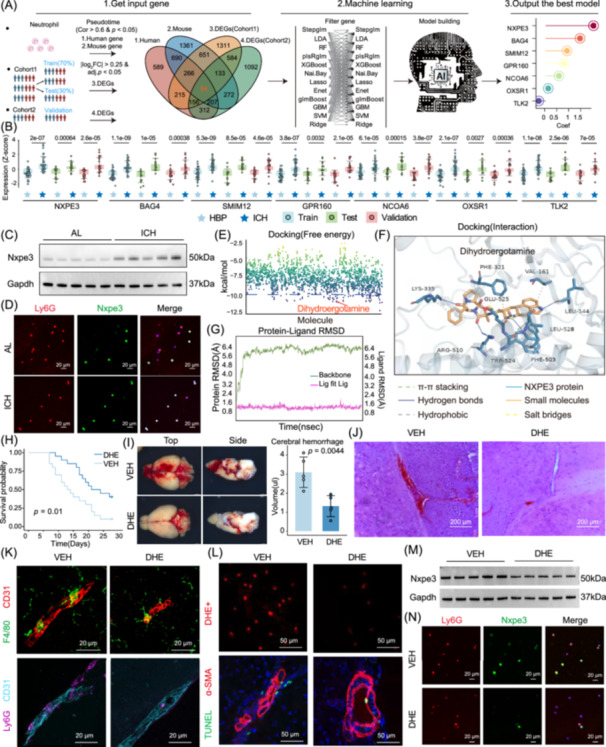
Identification of effective targets and drugs for patients with hypertensive intracerebral hemorrhage. (A) Machine learning integration framework. Cohort 1 was first divided into a 7:3 ratio for the training and testing sets, while cohort 2 was used as the validation set. Next, genes highly correlated with pseudotime in human and mouse single‐cell RNA sequencing (scRNA‐seq) data were intersected with differentially expressed genes between intracerebral hemorrhage (ICH) and high blood pressure (HBP) in both cohorts. This resulted in 64 genes, which served as input features. The optimal model was chosen by average area under the curve (AUC) from final test and validation sets, generating gene coefficient values. (B) Gene expression in bulk transcriptomic between HBP and ICH groups across training, test, and validation sets. (C) Elevated Nxpe3 protein expression in neutrophils from ICH mice as shown in western blot (WB) analysis. (D) Immunofluorescence (IF) reveals a higher proportion of Nxpe3+ neutrophils in ICH mice. (E) Molecular docking binding free energy analysis of drugs with NXPE3. (F) Interaction of dihydroergotamine (DHE) with NXPE3 in the binding pocket. (G) After 100 ns of molecular dynamics, root‐mean‐square deviation (RMSD) analysis showed the protein‐ligand system quickly reached equilibrium, with ligand RMSD around 1.2 Å, indicating stable binding. (H) Kaplan–Meier (KM) survival analysis of ICH incidence (*n* = 20). (I) Representative images of ICH in each group (left) and intracerebral hemorrhage quantification via hemoglobin assay (right). (J) Hematoxylin and eosin (H&E) stained images of ICH. (K) Upper panels: Immunostaining of F4/80+ macrophages around CD31+ vessels. Lower panels: Immunostaining of Ly6G+ neutrophils around CD31+ vessels. (L) Upper panels: Oxidative stress measured by DHE+. Lower panels: α‐SMA‐positive (α‐SMA⁺) vascular smooth muscle cell apoptosis. (M) WB showing decreased Nxpe3 protein in neutrophils after DHE treatment. (N) IF showing a reduced proportion of Nxpe3+ neutrophils in the DHE group versus control.

Machine learning has shown excellent performance in diagnosing risks across various non‐cancer diseases. It has also made significant progress in predicting responses to cancer immunotherapy [10, 11]. Although models exist to predict the onset of hypertension, there is still a significant gap in forecasting the risk of ICH in hypertensive patients. Studies suggest that ensemble approaches can improve the performance of machine‐learning models and outperform single‐model strategies [12]. In our study, patients were divided into training, testing, and validation sets. A multi‐model integration strategy was used to evaluate the predictive power of genes correlated with neutrophil differentiation (Pearson correlation > 0.6, *p* < 0.05) for ICH risk in hypertensive patients (Figure 2A). The Lasso + Stepglm[forward] model demonstrated the best performance, with an average area under the curve (AUC) of 0.977 in both the testing and validation sets (Figure S2A). This model identified seven key genes—*NXPE3*, *BAG4*, *SMIM12*, *GPR160*, *NCOA6*, *OXSR1*, and *TLK2* (Figure 2A)—all upregulated in patients with ICH (Figure 2B). *NXPE3*, exhibiting the highest coefficient in the model, appeared in 92 out of 134 models, underscoring its potential role in hypertension‐related ICH. Western blot (Figures 2C and S2B) and immunofluorescence (Figures 2D and S2C) confirmed significantly elevated NXPE3 expression in neutrophils from the ICH group.

### Dihydroergotamine can target NXPE3 and effectively alleviate the occurrence of ICH

NXPE3 was identified as a potential therapeutic target by calculating the binding free energy with 2110 FDA‐approved compounds, identifying 78 candidates with binding energies <−10 kcal/mol (Figures 2E and S2D). Focusing on dihydroergotamine (DHE), molecular docking revealed stable interactions with NXPE3, involving hydrogen bonds with LYS‐335, TRP‐524, PHE‐503, and LEU‐144, as well as π–π stacking and hydrophobic interactions (Figure 2F). A 100‐ns molecular dynamics simulation (Figure S2E) confirmed the stability of the DHE‐NXPE3 complex, with the DHE root‐mean‐square deviation remaining around 1.2 Å (Figure 2G) and minimal protein root‐mean‐square fluctuation variation (<4 Å, Figure S2F), indicating stable NXPE3 structure.

To validate DHE's therapeutic effect, it was tested in a spontaneous ICH mouse model. Although DHE, a treatment for migraines that mediates cerebrovascular constriction [13], did not affect systolic blood pressure (Figure S2H), it provided significant neuroprotection, reducing hemorrhage incidence (Figure 2H), hemorrhage volume (Figure 2I), and lesions (Figures 2J and S2I). Immunofluorescence analysis revealed decreased leukocyte infiltration (Figures 2K and S2J), and DHE reduced reactive oxygen species levels and vascular smooth muscle cell apoptosis (Figures 2L and S2K), preserving vascular integrity [14]. Furthermore, DHE treatment decreased NXPE3 expression in neutrophils (Figures 2M–N and S2L–M). These findings indicate that DHE may target NXPE3, potentially alleviating hypertension‐related ICH and immune‐inflammatory responses.

In summary, compared to previous studies [15, 16], our improved CCCF framework expands the range of scoring methods, reduces algorithmic biases, and enhances consensus properties. This facilitates the identification of phenotype‐related cells with high confidence and can be easily adapted for other studies. Additionally, the multi‐machine learning integration approach provides higher accuracy than methods relying on a few algorithms [17]. Large‐scale molecular simulations and preliminary phenotype validation support the clinical potential of NXPE3 as a therapeutic target. However, our study has limitations. The ICH model induces vascular damage but does not fully replicate the chronic, multifactorial progression of human hypertension, especially its long‐term effects on the brain and other organs. Further human studies are needed to confirm the clinical relevance of these findings. Moreover, the clinical translation of NXPE3 as a therapeutic target requires further investigation into its efficacy, safety, patient variability, and optimization of dosage and administration strategies.

## CONCLUSION

*NXPE3* is a promising biomarker for ICH patients, but further clinical validation is needed. Moreover, DHE has a high affinity for NXPE3 and can effectively alleviate the occurrence of ICH.

## AUTHOR CONTRIBUTIONS

**Meng Zhang**: Conceptualization; formal analysis; writing—original draft; writing—review and editing; funding acquisition. **Jingyuan Ning**: Methodology; software; formal analysis; writing—original draft; writing—review and editing. **Jing Liu**: Validation; investigation. **Yingying Sun**: Investigation; validation. **Ning Xiao**: Investigation; validation. **Haochen Xu**: Validation; formal analysis; data curation; writing—review and editing. **Jingzhou Chen**: Resources; writing—review and editing; project administration; funding acquisition.

## CONFLICT OF INTEREST STATEMENT

The authors declare no conflicts of interest.

## Supporting information


**Figure S1:** Multi‐omics Analysis Reveals the Immune Landscape of high blood pressure and hypertensive intracerebral hemorrhage.
**Figure S2:** Screening Early Warning Molecules for hypertensive intracerebral hemorrhage.

**Table S1:** Characteristics of cohort 1 and cohort 2.

## Data Availability

The data that supports the findings of this study are available in the supplementary material of this article. All data are available in the supplementary materials. The code supporting this study, along with bulk and single‐cell transcriptomic data, is available at https://github.com/AcetylCoALab/ICH. Supplementary materials (methods, figures, tables, graphical abstract, slides, videos, Chinese translated version and update materials) may be found in the online DOI or iMeta Science http://www.imeta.science/.
