# Supplementary material for: Peripheral blood immune landscape and NXPE3 as a novel biomarker for hypertensive intracerebral hemorrhage risk prediction and targeted therapy
Source: Imeta. 2025 Apr 11;4(3):e70030. doi: 10.1002/imt2.70030 (PMC12130563; doi:10.1002/imt2.70030)
Supplement: Supplementary file 1 — Figure S1: Multi‐omics Analysis Reveals the Immune Landscape of high blood pressure and hypertensive intracerebral hemorrhage. Figure S2: Screening Early Warning Molecules for hypertensive intracerebral hemorrhage. [file IMT2-4-e70030-s002.docx]

**Supporting information to Peripheral Blood Immune Landscape and NXPE3 as a Novel Biomarker for Hypertensive Intracerebral Hemorrhage Risk Prediction and Targeted Therapy**

**Running title: NXPE3 as a Biomarker for Hypertensive Intracerebral Hemorrhage**

Meng Zhang^# 1,2^, Jingyuan Ning^# 2^, Jing Liu^1^, Yingying Sun^1^, Ning Xiao^1^, Haochen Xu^* 1^, Jingzhou Chen^* 1,3^

^1^ State Key Laboratory of Cardiovascular Disease, Fuwai Hospital, National Center for Cardiovascular Diseases, Chinese Academy of Medical Sciences and Peking Union Medical College, Beijing, 100037, China

^2^ State Key Laboratory of Common Mechanism Research for Major Diseases & Department of Medical Genetics, Institute of Basic Medical Sciences & School of Basic Medicine, Chinese Academy of Medical Sciences & Peking Union Medical College, Beijing, 100005, China.

^3^ National Health Commission Key Laboratory of Cardiovascular Regenerative Medicine, Fuwai Central-China Hospital, Central-China Branch of National Center for Cardiovascular Diseases, Zhengzhou, 451464, China

^#^ These authors contributed equally: Meng Zhang, Jingyuan Ning

*Correspondence: [xuhaochen@fuwai.com](mailto:xuhaochen@fuwai.com) (Haochen Xu); [chendragon1976@aliyun.com](mailto:chendragon1976@aliyun.com) (Jingzhou Chen)

1. **Supplementary figures**

**Figure S1. Multi-omics Analysis Reveals the Immune Landscape of high blood pressure and hypertensive intracerebral hemorrhage.** (A) Principal component analysis (PCA) of cohort 1 and 2 after batch effect removal, showing no significant batch effects. (B) Marker gene expression for cell annotation. (C) No significant changes in cell proportions were observed in single-cell RNA sequencing (scRNA-seq). (D) Differential expression in mouse scRNA-seq; blue denotes downregulated, red upregulated genes. (E) Human scRNA-seq signatures in cellular classification consensus framework (CCCF) scored in bulk cohorts via single-sample gene set enrichment analysis (ssGSEA). (F) Neu_a signature gene expression in high- and low-risk neutrophils in human and mouse scRNA-seq.

**Figure S2. Screening Early Warning Molecules for hypertensive intracerebral hemorrhage.** (A) Area under the curve (AUC) of 134 machine learning models in training, test, and validation cohorts, ranked by average AUC values from the final testing and validation set. (B) Quantification of western blot (WB) results for Nxpe3. (C) Immunofluorescence (IF) quantification of Nxpe3+ neutrophil proportions. (D) Molecular docking of NXPE3 and dihydroergotamine (DHE), showing binding pose and pocket. (E) Construction of water box for protein-ligand system prior to 100 ns simulation. (F-G) After 100 ns of molecular dynamics, the root mean square fluctuation (RMSF) of the protein and ligand trajectories was analyzed. The protein's RMSF remained mostly stable, with fluctuations under 4 Å, except for the terminal residues. The ligand's RMSF was around 1 Å, indicating a stable protein-ligand binding. (H) Systolic blood pressure levels measured using noninvasive tail-cuff method (*n* = 10/group). (I) Intracerebral hemorrhage (ICH) distribution by size in each group (*n* = 5). (J) F4/80+ macrophage (left, *n* = 5) and Ly6G+ neutrophil immunostaining (right, *n* = 5) quantification. (K) DHE+ oxidative stress (left, *n* = 5) and α-SMA-positive (α-SMA⁺) vascular smooth muscle cell apoptosis immunostaining (right, *n* = 5) quantification. (L) WB quantification showing reduced Nxpe3 protein expression in peripheral blood neutrophils after DHE treatment. (M) IF quantification showing reduced Nxpe3+ neutrophils in the DHE-treated group compared to controls.

1. **Materials and Methods**

**Clinical data collection**

A total of 66 patients with ICH and 64 age- and sex-matched individuals with HBP were recruited from two independent cohorts for RNA sequencing between 2014 and 2019. In the first cohort, 46 patients with ICH and 46 patients with hypertension were enrolled at Cangzhou Central Hospital from 2014 to 2017. The second cohort consisted of 20 patients with ICH from the Affiliated Hospital of Hebei University and 18 patients with hypertension from the General Hospital of Ningxia Medical University. ICH diagnoses were confirmed by neurologists based on clinical history, physical examination, and imaging studies, including CT or MRI. Patients with hypertension were selected based on their medical histories, ensuring no prior history of stroke or cardiovascular events. Demographic and clinical data were collected *via* face-to-face surveys and by reviewing hospital or medical examination records. Exclusion criteria encompassed autoimmune diseases, cardiac diseases, liver diseases, renal diseases, cancer, and a history of stroke or cerebral infarction with hemorrhagic transformation. This study was reviewed and approved by the Human Ethics Committee of Fuwai Hospital (Approval No. 2016-732), adhering to the principles of Good Clinical Practice and the Declaration of Helsinki. Written informed consent was obtained from all participants or their legal representatives.

**Bulk transcriptome sequencing**

Total RNA was extracted using the RNeasy Mini Kit with on-column DNase step (Qiagen, Hilden, Germany) according to manufacturers’ protocol. Immediately following extraction, the total RNA concentration and A260:A280 ratio of each sample was determined via NanoDrop 2000 (Thermo Fisher Scientific, IL, USA). Then submitted to Annoroad Gene Technology Company Ltd (Beijing, China) for RNA sequencing. To ensure the quality of the data for subsequent analysis, raw sequencing data were initially processed with internal filtering procedures. The filtering criteria included: (1) removal of reads with adapter contamination (reads with more than five base pairs of adapter sequence; for paired-end sequencing, both ends of the read were discarded if one end was contaminated); (2) removal of low-quality reads (reads where more than 15% of bases had a quality score Q ≤ 19; for paired-end sequencing, both ends were discarded if one end was low quality); (3) removal of reads with more than 5% ambiguous bases (N bases; for paired-end sequencing, both ends of the read were discarded if one end had more than 5% N bases). Reference genome and gene annotation files were downloaded from the ENSEMBL database (<http://www.ensembl.org/index.html>). Clean data were aligned to the reference genome using HISAT2 (v2.2.1), and all count values were converted to transcripts per million (TPM), followed by log_2_(TPM+1) transformation. Batch effectsbetween the two cohorts were removed using the ComBat function from the sva package (v3.5.0) [1].

**Enrichment analysis**

Enrichment analysis of differentially expressed genes was conducted using the ClusterProfiler package [2] (v4.12.0). Enrichment was evaluated against databases from Gene Ontology (GO) (<http://geneontology.org/>), the Kyoto Encyclopedia of Genes and Genomes (KEGG) (<https://www.genome.jp/kegg/>), and Reactome (<https://reactome.org/>). Pathways with a false discovery rate (FDR)-adjusted *p*-value < 0.05 were considered significantly enriched. Visualization of the results was performed using the ComplexHeatmap package [3] (v2.20.0).

**Human single-cell sequencing sample collection**

In 2022, a cohort consisting of 7 patients with microbleeds and 6 age- and sex-matched hypertensive controls was recruited from the First Affiliated Hospital of Dalian Medical University for single-cell RNA sequencing. Patients with microbleeds were initially diagnosed based on pre-treatment blood pressure measurements, with systolic blood pressure (SBP) ≥ 160 mmHg and diastolic blood pressure (DBP) ≥ 100 mmHg. Diagnosis was further confirmed by neurologists using clinical examinations and medical history, with imaging revealing at least three microbleed lesions (2-5 mm in diameter, up to 10 mm, hypointense, oval or round) located in the basal ganglia. Exclusion criteria for patients with microbleeds included intracranial vascular malformations, cardiovascular diseases, moyamoya disease, subarachnoid hemorrhage, primary intraventricular hemorrhage, post-traumatic hemorrhage, cerebral infarction, liver diseases, kidney diseases, autoimmune disorders, cancer, and central nervous system infections. Patients with hypertension had similar initial diagnostic criteria (SBP ≥ 160 mmHg and DBP ≥ 100 mmHg) and shared exclusion criteria, with the additional exclusion of a history of stroke or hemorrhagic transformation of cerebral infarction. Demographic and clinical data were gathered through face-to-face interviews and review of hospital or examination records. This study was approved by the Human Ethics Committee of Fuwai Hospital (Approval No. 2022-1692) and conducted in compliance with Good Clinical Practice and the Declaration of Helsinki. Written informed consent was obtained from all participants or their legal representatives.

**Single-cell sequencing process for human samples**

Transcriptomic data from single cells were obtained using the BD Rhapsody system. Single-cell capture was accomplished by dispersing a single-cell suspension into over 200,000 microwells using a limiting dilution method. Oligonucleotide-barcode-coated beads were introduced to ensure that each microwell contained one cell and one bead. Following cell lysis, polyadenylated RNA molecules were captured by the beads, which were subsequently collected for reverse transcription. During cDNA synthesis, each cDNA molecule was tagged with a unique molecular identifier (UMI) at the 5' end (corresponding to the 3' end of the mRNA transcript) and a cell-specific label. Whole transcriptome libraries were generated *via* the BD Rhapsody single-cell whole-transcriptome amplification workflow, which involved second-strand cDNA synthesis, ligation of the WTA adaptor for universal amplification, and 18 cycles of PCR to amplify the adaptor-ligated cDNA. Sequencing libraries were constructed using random priming PCR to enrich the 3' ends of transcripts associated with cell labels and UMIs. Libraries were quantified using a High Sensitivity DNA chip (Agilent) on a Bioanalyzer 2200 and the Qubit High Sensitivity DNA assay (Thermo Fisher Scientific). Sequencing was performed on an Illumina sequencer (Illumina, San Diego, CA) using a 150 bp paired-end protocol.

**Animal model of spontaneous intracerebral hemorrhage**

Male wild-type C57BL/6N mice, aged 8 months (28-34 g), were obtained from the National Resource Center of Model Mice. The mice were housed in a specific pathogen-free environment under a 12-hour light/dark cycle and were provided with a standard rodent diet. This study comprised three cohorts of mice. The first cohort, termed AL, was used to generate a hypertension model with a risk of cerebral hemorrhage. Hypertension was induced by administering angiotensin II (AngII; 1000 ng/kg per min; Sigma-Aldrich, MO, USA) *via* subcutaneously implanted osmotic pumps (Durect Corporation, CA, USA) under anesthesia with 2% isoflurane in an O_2_ mixture (1 L/min). On the day of pump implantation, L-NAME (100 mg/kg per day; Sigma-Aldrich, MO, USA) was introduced into the drinking water. Systolic blood pressure was measured on day 3 post-implantation using tail-cuff plethysmography (BP-2010A, Softron, Japan). Peripheral blood was collected and subjected to red blood cell lysis for single-cell sequencing. The second cohort was used to generate a spontaneous ICH model. Details of the animal model and experimental stroke assessment have been described previously.4 Transient acute hypertension was induced one week after pump implantation by daily AngII injections (0.5 mg/kg, s.c., twice daily from days 7 to 28 post-implantation). Systolic blood pressure was monitored bi-weekly. Clinical stroke signs, including circling behavior, contralateral forelimb extension, and other motor dysfunctions, were evaluated through daily neurological examinations. Upon observation of stroke symptoms, peripheral blood was collected for single-cell sequencing following red blood cell lysis. The third cohort was utilized to assess the effects of NXPE3-targeted compounds on hypertensive ICH. A hypertensive ICH model was established as described for the second cohort, with the addition of daily intraperitoneal injections of dihydroergotamine (1.0 mg/kg, HY-B0670A, MCE, USA) starting two weeks prior to model induction. Upon the appearance of stroke symptoms, mouse brains were collected for further analysis. All animal experiments were approved by the Committee of Fuwai Hospital on Ethics of Animal Experiments (Approval No: FW-2020-12) and were conducted in compliance with the National Institutes of Health’s Guide for the Care and Use of Laboratory Animals. The study adhered to the ARRIVE (Animal Research: Reporting of *In Vivo* Experiments) guidelines for reporting animal experiments.

**Hematoxylin-eosin staining and quantification of hemorrhage**

Brains were fixed overnight in 4% paraformaldehyde at 4°C, followed by sequential dehydration in 20% and 30% sucrose solutions, each at 4°C overnight. Coronal sections, each 20 μm thick, were prepared for histological analysis. For hematoxylin-eosin (H&E) staining, sections were incubated in hematoxylin for 2 minutes and eosin for 1 minute. Every fifth serial section was analyzed to assess the size and number of ICHs throughout the brain. Images of ICHs were captured using a Leica DM6000B microscope, and hemorrhages larger than 10^–6^ mm^3^ in area were quantified using ImageJ software. Hemorrhage size was calculated by multiplying the ICH area in each section (mm^2^) by the section interval (20 × 10^3^ mm). For hemorrhage quantification, 500 μl of Drabkin reagent (Sigma-Aldrich, MO, USA) was added to homogenized hemispheric brain tissue, followed by centrifugation at 13,000 rpm for 30 minutes. The optical density of the supernatant was measured at 540 nm using a spectrophotometer (Thermo Fisher Scientific, IL, USA), and hemorrhage volume was quantified based on a reference curve established in prior studies.

**TUNEL staining**

Terminal deoxynucleotidyl transferase-mediated dUTP nick-end labeling (TUNEL) (Sigma-Aldrich, MO, USA) was performed using an *in situ* cell death detection kit. Tissue sections were incubated with primary α-SMA antibodies, followed by secondary antibodies, and then with the TUNEL reaction mix according to the manufacturer’s instructions. The number of TUNEL-positive (TUNEL+) cells within α-SMA-positive arteries was quantified using ImageJ software.

Frozen tissue sections were immunostained using the following primary antibodies: goat anti-CD31 (AF3628, R&D Systems, MN, USA), anti-actin α-smooth muscle-Cy3™ (αSMA, clone 1A4, C6198, Sigma-Aldrich, USA), rat anti-Ly6G (551459, BD Pharmingen, USA), and anti-F4/80 (ab6640, Abcam, MA, USA). Secondary antibodies included Alexa Fluor 594-conjugated donkey anti-goat IgG, Alexa Fluor 594-conjugated donkey anti-rat IgG, and Alexa Fluor 488-conjugated donkey anti-rat IgG. Imaging was performed using a Leica SP8 laser-scanning confocal microscope and a Leica DM6000B microscope. For quantification, images of all markers were captured from four random fields in three sections per sample (with coordinates 1.2 mm, 2.2 mm, and 3.2 mm posterior to bregma) at 40× magnification. Five mice per group were analyzed.

**Single-cell sequencing process for mouse samples**

Single-cell RNA sequencing was conducted using the Chromium Single Cell 3’ GEM, Library & Gel Bead Kit v3.1 (10x Genomics, PN-1000268). A single-cell suspension, containing 700-1200 viable cells per ml as determined by the CellDrop FL Cell Counter, was loaded onto a Chromium Single Cell Chip (Chromium Single Cell G Chip Kit, 10x Genomics, PN-1000120), following the manufacturer's protocols. The target capture rate aimed for 10,000 individual cells per sample. After cell capture, the cells were lysed, and the released RNA was barcoded through reverse transcription within individual gel beads in the emulsion. Complementary DNA (cDNA) synthesis and amplification occurred in each droplet using a T100 PCR Thermal Cycler (Bio-Rad) with the following conditions: 45 minutes at 53°C, 5 minutes at 85°C, and a final hold at 4°C. The concentration and quality of cDNA were assessed using a Qubit Fluorometer (Thermo Scientific) and Bioanalyzer 2100 (Agilent), respectively. Single-cell RNA sequencing libraries were prepared and sequenced to a depth of 75,000 reads per cell on an Illumina platform by Annoroad Gene Technology Co., Ltd. FASTQ files were processed using the Cell Ranger (v6.1.2) count pipeline with the Mus_musculus GRCm38 mouse reference genome to generate feature-barcode matrices.

**Single-cell data quality control**

Quality control of the single-cell RNA sequencing data was performed using standard procedures in the R environment (v4.4.0). The count matrix was read with the Read10X function from the Seurat [4] package (v4.4.0) and converted to the dgCMatrix format. Individual samples were merged into a single aggregate object using the merge function, and cell labels were made unique using the RenameCells function. Doublets were removed using the Scrublet [5] algorithm (v0.2.3). Low-quality cells were filtered based on the following criteria: genes expressed in fewer than 200 cells were excluded, and cells with fewer than 500 or more than 4,000 expressed genes were removed. Gene expression was normalized across cells using the global-scaling normalization method (LogNormalize), with a scale factor of 10,000. The top 2,000 variable genes, identified using the FindVariableFeatures function, were selected for further analysis. To correct for unwanted sources of variation, the ScaleData function was applied, regressing out UMI counts and percent mitochondrial content. Dimensionality reduction was performed using principal component analysis (PCA) on highly variable features, selecting the first 30 principal components (PCs) for analysis. Batch effects between samples were mitigated using the Harmony method (v0.1.1). Cells were visualized in reduced dimensions using uniform manifold approximation and projection (UMAP). Clustering analysis was performed based on edge weights between cells. A shared nearest-neighbor graph was constructed using the Louvain algorithm, implemented through the FindNeighbors and FindClusters functions, with the resolution parameter tested between 0.1 and 1. The clustree (v0.5.1) was used to visualize clustering outcomes at various resolutions, with the most distinct clustering results observed at a resolution of 0.3. Cell clusters were annotated by identifying differentially expressed markers using the FindAllMarkers function. Annotations were based on cell markers, relevant literature, and the cell taxonomy database (<https://ngdc.cncb.ac.cn/celltaxonomy/>).

**Identification of phenotype-associated cells**

Gene signature sets, derived from gene expression analyses across various cell types or biological states, are pivotal constructs in biological research. GSEA serves as an effective method to associate these signature sets with previously uncharacterized clusters, with the degree of enrichment indicating the strength of the association between the biological function represented by the gene set and the given cluster. In single-cell sequencing, utilizing gene signature sets to delineate distinct cell types and states within unknown clusters has proven to be a powerful approach for elucidating biological functions [6,7]. Additionally, several cell classifiers have been developed based on curated gene sets, such as IKarus, Cancer-Finder, ScType, scPriorGraph, sc-ImmuCC, SenCID, and TCellSI. These classifiers partition cells based on gene feature scores. However, mainstream functional class scoring methods—such as AUCell, UCell, and JASMINE—differ in their capabilities to score and identify cellular characteristics [8]. Consequently, relying on a single scoring method may limit the depth of insights; integrating multiple methods has been shown to enhance performance [9]. In this study, to identify neutrophils associated with the high-risk phenotype of ICH, a novel Cellular Classification Consensus Framework (CCCF) was developed by refining existing approaches (Figure 1E) [10]. Firstly, differential expression analysis of neutrophils in human single-cell sequencing data was performed using the Seurat package (v4.4.0) with the FindMarkers function. Genes with |log_2_FC| > 0.25 and adjusted p < 0.05 were considered differentially expressed in the ICH group. Upregulated genes were designated as signature1, and downregulated genes as signature2. Gene set scoring for each cell was performed using the irGSEA [8] package (v3.2.6), calculating signature1 and signature2 scores for each cell. If the signature1 score was greater than the median value, it was assigned a value of 1; otherwise, it was assigned a value of 0. Conversely, if the signature2 score exceeded the median, it was assigned a value of 0, and 1 otherwise. Given that 19 gene set scoring algorithms were utilized, each cell received a total of 38 scores—19 for signature1 and 19 for signature2. Finally, the results of the cell assignments were summed for each cell. Cells were classified based on the sum of these scores: a total score of 38 indicated high-risk neutrophils, while a score of 0 denoted low-risk neutrophils. Cells with intermediate scores were excluded from further analysis. This CCCF methodology, adapted for mouse single-cell sequencing based on human signatures, utilized the nichenetr [11] package (v2.2.0) for species-specific gene symbol conversion, enhancing the accuracy of phenotype classification in single-cell transcriptomic data. To validate the bulk transcriptome data, a quantitative comparison of signatures was performed using ssGSEA analysis, a method widely applied in bulk analyses.

**Pseudotime Analysis**

The scTour [12] (v1.1.0) method provides an innovative and integrated framework for analyzing cell dynamics using single-cell genomics datasets. Operating within a Python environment (v3.12.7), the software offers a comprehensive approach to depicting developmental processes from multiple perspectives, including pseudotime, vector fields, and latent spaces. This method extends its capabilities into a multi-task architecture, utilizing raw count matrices as input to perform dataset-internal inference in a batch-sensitive manner. scTour incorporates two key parameters that balance reconstruction errors from both the encoder-derived latent space and the ODE-solver-derived latent space. A smaller value, which assigns a lower weight to the reconstruction error from the encoder-derived latent space, generally produces latent representations that order cells based on pseudotime, but it may face challenges distinguishing between cell types with similar developmental trajectories. In our analysis, the default parameter value of 0.5 was used. The get_time function was subsequently employed to model cellular differentiation trajectories, and a UMAP embedding was generated based on the inferred latent space for visualization.

Transcription factor activity was assessed using the SCENIC [13] (v1.3.1) approach. Initially, GENIE3 (v1.26.0) was applied to construct a regulatory network between transcription factors and their potential target genes based on co-expression patterns. This network was refined by integrating motif relationships and ranking motif-gene regulatory potential, resulting in a refined network referred to as a "regulon." The regulon includes target genes that are directly bound by transcription factors through upstream motifs. To evaluate the activity of each regulon across all cells, AUCell (v1.26.0) was employed.

**Construction of machine learning predictive models**

Machine learning has shown exceptional performance in risk diagnosis for various non-cancer diseases and has made significant strides in predicting responses to cancer immunotherapy. Although models have been developed to predict the onset of hypertension, there remains a notable gap in forecasting the risk of ICH among patients with hypertension. Research has indicated that ensemble methods can enhance the overall performance of machine learning models, often surpassing single-model approaches. In this study, twelve machine learning algorithms were employed to construct binary classification models, including stepwise regression (stepglm), linear discriminant analysis (LDA), random forest (RF), partial least squares regression generalized linear model (plsRglm), extreme gradient boosting (XGBoost), naive Bayes (naiveBayes), least absolute shrinkage and selection operator (Lasso), elastic net regression (Enet), generalized linear model by likelihood-based boosting (glmBoost), gradient boosting machine (GBM), support vector machine (SVM), and ridge regression (Ridge). All algorithms were trained on the same preprocessed dataset to ensure consistency and comparability of results. Data were standardized prior to model training to eliminate scale discrepancies, adjusting the mean to zero and the standard deviation to one.

To ensure model accuracy and robustness, hyperparameters for each algorithm were carefully tuned, and performance was evaluated using cross-validation. The area under the curve (AUC) was used as the metric for model performance, with the best-performing model selected based on the highest average AUC across training, testing, and validation cohorts. For Elastic Net and Lasso regression, 10-fold cross-validation (cv.glmnet) was utilized to select the regularization parameter, lambda, which controls the strength of regularization. Larger values of lambda enforce stronger regularization, thus preventing overfitting, while smaller values allow for a better fit to the data. In Elastic Net, the alpha parameter controls the balance between Lasso regression (*alpha* = 1) and Ridge regression (*alpha* = 0), and the model was optimized by adjusting alpha between 0 and 1. In Lasso regression, alpha was fixed at 1. Stepwise regression (stepglm) was performed using a stepwise selection method, with both forward and backward selection, guided by the Akaike Information Criterion (AIC). The model with the lowest AIC was selected as the optimal model. For SVM, recursive feature elimination (RFE) was employed to identify the optimal feature subset. RFE iteratively trains the SVM model on various subsets of features, selecting the subset that yields the best performance. Key parameters for SVM, including the cost (penalty parameter) and gamma (kernel parameter), were optimized using cross-validation to achieve the optimal SVM model. For RF and GBM, model optimization was carried out by adjusting critical parameters such as the number of trees (ntree), the number of features selected per tree (mtry), the maximum depth of trees (max_depth), and the minimum number of samples per leaf node (nodesize). These parameters were fine-tuned using cross-validation to mitigate overfitting and enhance model generalization. For GBM, particular attention was given to the learning rate (shrinkage), which controls each tree's contribution to the final model. A smaller learning rate typically improves model stability but requires a higher number of trees for adequate fitting. Cross-validation was employed to determine the optimal values for the number of trees, tree depth, and learning rate.

XGBoost, a highly efficient boosting algorithm, was optimized by adjusting parameters such as the learning rate (eta), tree depth (max_depth), number of iterations (nrounds), and subsample ratio (subsample). Cross-validation was utilized to identify the optimal combination of these parameters. For glmBoost, which combines generalized linear models with boosting, the step size and maximum iterations were optimized to prevent overfitting and improve model performance. LDA was optimized through the train function with cross-validation to select the most effective model. Naive Bayes, based on Bayesian theory, assumes feature independence and computes class probabilities accordingly, selecting the class with the highest probability as the predicted outcome.

After training all the models, their performance was evaluated by computing classification results for the training, testing, and validation datasets using the predict function. Model performance was assessed by calculating the AUC, with higher AUC values indicating superior classification performance. The final model was selected based on the average AUC values from the test and validation cohorts, and models were ranked according to their AUC performance. The best-performing model was subsequently chosen for further analysis and application.

**Molecular docking**

Molecular docking analysis was performed to predict the binding modes and affinities of ligand molecules to the target protein NXPE3. The 3D structure of NXPE3 was retrieved from the AlphaFold database (https://alphafold.ebi.ac.uk/entry/Q969Y0), which provides high-confidence predicted protein structures. Prior to docking, the protein structure was prepared by removing any heteroatoms, such as bound ligands or cofactors, and water molecules that could interfere with the docking process. Hydrogen atoms were then added to the structure to ensure proper protonation states, facilitating accurate interactions with the ligands. The library of FDA-approved small molecules was obtained from the ZINC15 database (https://zinc15.docking.org/), which offers a vast collection of commercially available compounds in mol2 format. This library was selected to prioritize compounds with potential therapeutic applications. Each ligand was optimized using the MM2 force field, commonly employed for energy minimization and molecular structure optimization. After optimization, ligands were converted from mol2 to pdbqt format using Open Babel (v3.1.1), a widely used tool for chemical file format interconversion, ensuring compatibility with AutoDock Vina (v1.2.3).

Given that NXPE3 lacks a defined active site in the literature and has limited structural information available, a blind docking approach was employed. The docking grid was set to cover the entire surface of the protein, allowing exploration of all potential binding regions. This approach compensates for the absence of prior knowledge regarding the exact binding site, ensuring that all possible binding sites are considered during the docking process. Docking simulations were performed using AutoDock Vina, a well-established molecular docking program that predicts the binding affinity and orientation of ligands in the receptor’s binding site. AutoDock Vina utilizes a semi-empirical scoring function and efficient search algorithms to predict the most likely binding poses of the ligands, with a focus on binding energy and geometric complementarity between the ligand and receptor. A lower binding free energy indicates a stronger, more stable interaction. The results were analyzed to identify the most favorable binding poses, with the best binding affinity serving as an indicator of the strongest ligand-protein interaction. The docking results were further validated by comparing the binding affinities of the selected ligands with known protein-ligand binding data, when available, and through visualization of the docking poses using molecular visualization software such as PyMOL (v.2.3).

**Molecular dynamics**

Molecular dynamics (MD) simulations were employed to examine the detailed fluctuations and conformational changes of the protein. To monitor the dynamic behavior of the system, the root-mean-square deviation (RMSD) of the α-carbon atoms relative to the initial structure was calculated, providing insight into the stability and alterations in the protein’s conformation during the simulation. To further evaluate the stability of the binding mode between the protein and the ligand, conventional MD simulations of the protein-ligand complex were performed using the Desmond module from the Schrödinger (v2021) Drug Design Suite. The MD simulation setup was designed based on the protein-ligand system. The protonation states of the protein's amino acid residues were determined using PROPKA 3.0, a tool that predicts protonation states of titratable residues at physiological pH. All initial crystallographic water molecules from the selected protein structure were retained to maintain the system’s fidelity to the natural protein environment. The initial all-atom complex model was constructed using Schrödinger's System Builder module. The OPLS_4 force field was applied to parameterize both the protein and the small molecules, while the TIP3P water model was used for solvation. The system was enclosed in a cubic water box with a 10.0 Å boundary thickness to ensure proper solvation. The complex was neutralized by adding sodium or chloride ions, as needed, to achieve charge balance.

Following system setup, a series of MD simulations were conducted using standard simulation parameters. Initially, the system's energy was minimized for 50,000 steps using the steepest descent method to remove any steric clashes or unfavorable interactions. After energy minimization, the positions of the heavy atoms were constrained, and two equilibration phases were performed: the NVT ensemble (constant volume and temperature) and the NPT ensemble (constant pressure and temperature), each for 50,000 steps. The temperature was maintained at 300 K using the Langevin thermostat, and the system pressure was regulated at 1 atm using the Monte Carlo barostat. These equilibration steps ensured system stability before the production run. Once the system had equilibrated, an unconstrained production run lasting 100 ns was executed to simulate the dynamics of the protein-ligand complex. During this phase, the system’s energy and coordinates were recorded every 10 ps to create a trajectory for subsequent analysis. Post-simulation, the resulting trajectories were analyzed to evaluate the structural stability and dynamic behavior of the complex. Schrödinger 2023 was utilized to generate interaction maps and dynamic trajectory animations, offering a visual representation of the protein-ligand interactions throughout the simulation. The analysis specifically focused on calculating RMSD, root-mean-square fluctuation (RMSF), and secondary structure content throughout the 100 ns MD simulation. RMSD was computed to monitor the overall conformational stability of the complex over time, while RMSF was used to assess the flexibility of individual residues, providing insights into regions of the protein that undergo significant fluctuations. Additionally, changes in the protein’s secondary structure were tracked to evaluate how ligand binding might influence protein folding and stability. This comprehensive MD simulation approach facilitated the evaluation of both the stability of the protein-ligand interaction and the dynamic behavior of the protein, yielding essential insights into the potential binding modes and the protein's flexibility upon ligand binding.

**Assessment of Nxpe3 Expression in Neutrophils from AL and ICH Mice Using Western Blot and Immunofluorescence**

For the investigation of Nxpe3 expression, plasma samples (1000 μL) were collected from AL and ICH mice through retro-orbital blood sampling (*n* = 5 per group). In a separate experiment, mice were treated with either VEH or DHE (1.0 mg/kg, HY-B0670A, MCE, USA) for two weeks prior to AL induction. On day 5 post-induction, plasma samples (1000 μL) were again collected *via* retro-orbital blood sampling (*n* = 5 per group). Neutrophils were isolated from plasma using the Neutrophil Isolation Kit (Miltenyi, 130-097-658), following the manufacturer’s protocol. The purified neutrophils were subsequently lysed for Western blot (WB) analysis, and a portion was cytospun onto slides for immunofluorescence (IF) staining. The following primary antibodies were employed: mouse anti-NXPE3 (MA5-32894, Invitrogen, USA) and rat anti-Ly6G (551459, BD Pharmingen, USA).

**Statistical Analysis**

A *t-test* was applied to analyze group differences for normally distributed variables, with the Mann–Whitney *U* test used for non-normally distributed variables. Differences in categorical variables were assessed using Pearson's chi-squared test or Fisher's exact test. Kaplan-Meier analysis was used to generate survival curves, and significance was determined using the log-rank test. A *p*-value of less than 0.05 was considered statistically significant. All analyses were two-sided and performed using R software (v4.4.0).

**Reference**

1. Leek, Jeffrey T., W. Evan Johnson, Hilary S. Parker, Andrew E. Jaffe-John D. Storey. 2012. “The sva package for removing batch effects and other unwanted variation in high-throughput experiments.” Bioinformatics 28: 882-883. https://doi.org/10.1093/bioinformatics/bts034

2. Wu, Tian-zhi, Er-qiang Hu, Shuang-bin Xu, Mei-jun Chen, Ping-fan Guo, Ze-han Dai, Ting-ze Feng, et al. 2021. “clusterProfiler 4.0: A universal enrichment tool for interpreting omics data.” The Innovation 2: 100141. https://doi.org/https://doi.org/10.1016/j.xinn.2021.100141

3. Gu, Zu-guang. 2022. “Complex heatmap visualization.” iMeta 1: e43. <https://doi.org/https://doi.org/10.1002/imt2.43>

4. Hao, Yu-han, Stephanie Hao, Erica Andersen-Nissen, William M. Mauck, Shi-wei Zheng, Andrew Butler, Maddie J. Lee, et al. 2021. “Integrated analysis of multimodal single-cell data.” Cell 184: 3573-3587.e3529. https://doi.org/https://doi.org/10.1016/j.cell.2021.04.048

5. Wolock, Samuel L., Romain Lopez-Allon M. Klein. 2019. “Scrublet: Computational Identification of Cell Doublets in Single-Cell Transcriptomic Data.” Cell Systems 8: 281-291.e289. https://doi.org/https://doi.org/10.1016/j.cels.2018.11.005

6. Zhang, Ting-ting, Fa-ming Zhao, Ya-hang Lin, Ming-sheng Liu, Hong-qing Zhou, Feng-zhen Cui, Yang Jin, Liang Chen, Xia Sheng. 2024. “Integrated analysis of single-cell and bulk transcriptomics develops a robust neuroendocrine cell-intrinsic signature to predict prostate cancer progression.” Theranostics 14: 1065-1080. https://doi.org/10.7150/thno.92336

7. Hara, Toshiro, Rony Chanoch-Myers, Nathan D. Mathewson, Chad Myskiw, Lyla Atta, Lillian Bussema, Stephen W. Eichhorn, et al. 2021. “Interactions between cancer cells and immune cells drive transitions to mesenchymal-like states in glioblastoma.” Cancer Cell 39: 779-792.e711. https://doi.org/https://doi.org/10.1016/j.ccell.2021.05.002

8. Fan, Chui-qin, Fu-yi Chen, Yuan-guo Chen, Liang-ping Huang, Man-na Wang, Yu-lin Liu, Yu Wang, et al. 2024. “irGSEA: the integration of single-cell rank-based gene set enrichment analysis.” Briefings in Bioinformatics 25: https://doi.org/10.1093/bib/bbae243

9. Alhamdoosh, Monther, Milica Ng, Nicholas J Wilson, Julie M Sheridan, Huy Huynh, Michael J Wilson-Matthew E Ritchie. 2016. “Combining multiple tools outperforms individual methods in gene set enrichment analyses.” Bioinformatics 33: 414-424. https://doi.org/10.1093/bioinformatics/btw623

10. Zhang, Yan-ru, Bo Zhang, Cheng-qian Lv, Nan Zhang, Kai-yuan Xing, Zi-xuan Wang, Rong-kai Lv, Ming-chen Yu, Chao-han Xu, Yi-han Wang. 2023. “Single-cell RNA sequencing identifies critical transcription factors of tumor cell invasion induced by hypoxia microenvironment in glioblastoma.” Theranostics 13: 3744-3760. https://doi.org/10.7150/thno.81407

11. Browaeys, Robin, Wouter Saelens-Yvan Saeys. 2020. “NicheNet: modeling intercellular communication by linking ligands to target genes.” Nature Methods 17: 159-162. https://doi.org/10.1038/s41592-019-0667-5

12. Li, Qian. 2023. “scTour: a deep learning architecture for robust inference and accurate prediction of cellular dynamics.” Genome Biology 24: 149. https://doi.org/10.1186/s13059-023-02988-9

13. Aibar, Sara, Carmen Bravo González-Blas, Thomas Moerman, Vân Anh Huynh-Thu, Hana Imrichova, Gert Hulselmans, Florian Rambow, et al. 2017. “SCENIC: single-cell regulatory network inference and clustering.” Nature Methods 14: 1083-1086. https://doi.org/10.1038/nmeth.4463
